# Incidence of Sickle Cell Disease and Other Hemoglobin Variants in 10,095 Lebanese Neonates

**DOI:** 10.1371/journal.pone.0105109

**Published:** 2014-09-02

**Authors:** Evelyne Khoriaty, Rim Halaby, Mohamad Berro, Ahmad Sweid, Hussein A. Abbas, Adlette Inati

**Affiliations:** 1 Department of Pediatrics, State University of New York, Syracuse, New York, United States of America; 2 School of Medicine, Lebanese American University, Byblos, Lebanon; 3 School of Medicine, American University of Beirut Medical Center, Beirut, Lebanon; 4 Lebanese American University and Division of Pediatric Hematology-Oncology, Rafic Hariri University Hospital, Beirut, Lebanon; Brighton and Sussex Medical School, United Kingdom

## Abstract

Hemoglobinopathies are highly prevalent diseases and impose a public health burden. Early diagnosis and treatment can ameliorate the course of these diseases and improve survival. Despite purported high incidence of hemoglobinopathies in Lebanon, there are no nationwide screening programs. In this study, newborn screening utilizing high pressure liquid chromatography was executed in all public hospitals across Lebanon between 2010 and 2013. All newborns with an abnormal hemoglobin (Hb) were offered genetic counseling and all those with disease were enrolled in comprehensive hemoglobinopathy clinics. Among newborns, 2.1% were found to have an abnormal Hb variant with sickle Hb being the most common while 0.1% were found to have sickle cell disease (SCD). The majority of those with SCD had non-Lebanese origins. The most common causes of hospitalizations in infants with SCD were acute splenic sequestration and pain crises. No bacteremia or other life threatening infections were noted. At a median follow up 14 months (follow up range 7 to 34 months), all children with disease are alive and compliant with treatment. Systematic screening for SCD and other Hb variants was shown to be feasible, cost effective, and of accurate predictive value. This program was also clinically effective because it led to the identification of babies with disease and to providing them with free early multidisciplinary care. Conclusively, a newborn screening program should be implemented across Lebanon to detect hemoglobinopathies and initiate early therapeutic and preventive strategies and genetic counseling.

## Introduction

Hemoglobinopathies are highly prevalent hereditary disorders of hemoglobin (Hb) characterized by the presence of an abnormal β-globin chain (hemoglobin variant as in sickle cell disease) or a decrease or absence of α - or β -globin chains (thalassemias). The sickle hemoglobin (HbS) is the most common hemoglobin variant resulting from an amino acid substitution of valine for glutamic acid at position 6 of the adult *β*-globin chain. Individuals affected by sickle cell disease (SCD) have two copies of *HbS*, or one copy of *HbS* along with another Hb variant such as *HbC* or HbD, *HbO* ^Arab^ or *Hbβ* thalassemia [1], [2]. The resultant mutant Hb leads to a wide range of manifestations including hemolytic anemia, vaso-occlusive crises and tissue infarctions, life threatening infections and end organ failure. [3], [4].

Newborn screening (NBS) coupled with early prophylactic penicillin, immunizations, comprehensive care, and parental education has been shown to significantly reduce SCD morbidity and mortality [5], [6]. The main objectives of NBS for haemoglobinopathies are 1.Detection and treatment of neonates affected with SCD and 2. Detection of carriers and provision of genetic counseling to their families. In countries with high disease prevalence as the Mediterranean region, it is prudent to initiate NBS programs to detect Hb disorders.

In Lebanon, epidemiological data on SCD and national guidelines on clinical management are lacking. The mandatory premarital blood testing does not include screening for sickle cell disease or carrier state. In addition, there is no NBS program for hemoglobinopathies and affected patients are diagnosed when disease complications start to manifest or when they have an affected sibling. Therefore, the exact incidence of SCD and other Hb variants in Lebanon is currently unknown and needs to be determined.

The primary objective of this study was to assess the birth incidence of SCD and other Hb variants in public hospitals in Lebanon. The secondary objectives were initiating appropriate prophylactic and therapeutic measures in afflicted individuals and providing genetic counseling for heterozygote neonates. Moreover, this study aimed at providing evidence to Lebanese health policy makers for the importance of integrating screening for SCD in premarital and neonatal registration programs.

## Materials and Methods

### Population and Study Design

This is a prospective cross-sectional study conducted in all Lebanese public hospitals between December 2010 and March 2013. Mothers were informed about the testing method, risks of phlebotomy, benefit of NBS and early treatment by trained personnel and through Arabic written pamphlets. After maternal consent, blood from heels of newborns was drawn on a filter paper and sent to the screening central lab. Maternal verbal consent was received before drawing blood. The participant's consent was documented in the medical chart. The newborn screening program was supported by the Ministry of Health (MOH) in Lebanon and was executed in the Lebanese public hospitals, which are managed by the MOH. The ethics committees at the MOH and the respective hospitals approved the verbal consent procedure. All tests were done free of charge. Institutional Review Board approval was received in all hospitals: Karantina, Sir elDenyyeh, Abdalla AlRassi, AlShahhar, Sibline, Sour, AlNabatieh, Mays AlJabal, Hasbayya, Marjeyoun, Hermal, Baalback, Dahr AlBachek, Saida, Rafic Hariri University Hospital, Tripoli General Hospital, Zahle Hospital.

### Data Collection

Socio-demographic and gestational data on all newborns were collected and documented on paper and computer database. Data collected included information on name, parents telephone number, regional and religious distribution, consanguinity, family history of hemoglobinopathies, gender, type of delivery, neonatal complications, first and confirmatory tests collection dates, first and confirmatory HPLC results and reason of failure of confirmatory testing.

### High-performance liquid chromatography (HPLC)

HPLC analysis was performed on heel stick dry blood samples in a central lab experienced in NBS and diagnosis of hemoglobinopathies. HPLC technique separates the various Hb fractions, gives qualitative determinations of Hb variants on the basis of their respective retention times and quantifies Hb A2 and Hb F. Based on Hb type assessment, sickle-cell/other Hb variant carriers or homozygous/double heterozygote patients were identified.

### Reporting of results and follow up

Positive results were conveyed to the principal investigator or the sub investigators and to the nursing staff of the maternity service concerned. Parents of all newborns with Hb variants were contacted within 8 weeks of receiving screening results and asked to bring their children for repeat testing. Upon confirmation of SCD and or other Hb variant disease, patients were enrolled at no cost in 2 SCD comprehensive clinics and cared for by a multidisciplinary team. Genetic counseling was provided to the parents of all carriers and those with disease. When resources were available, parents and siblings were also offered testing for SCD and other Hb variants.

### Data Analysis

The data were analyzed using the IBM Statistical Package for Social Sciences version 19. Sample characteristics were reported using descriptive statistics. Normality of the data was checked through standardized skew statistics, and the Levene statistic. Pearson's correlation coefficient (r) was used to examine variable associations. Groups were compared on the respective study variables through the t statistic for continuous and normally distributed dependent variables and chi squares as well as the Kruskal Wallis test were used for categorical variables. Finally the Mann Whitney U test was used to assess group differences on relevant variables. Statistical significance was set at the 95^th^ percentile and two tailed statistics were applied throughout.

## Results

Our database included information on a total of 10,095 (50.2% males and 49.8% females) newborns. Two hundred and eleven (2.1%) screened newborns had a Hb variant and 97.9% had a normal Hb profile (Table 1). One hundred and ninety seven (93%) were carriers of a Hb variant while 14 (7%) had hemoglobinopathies. The most common Hb variant detected was HbS (84.4%) followed by HbD (10.5%), HbC (2.8%), *HbO* ^Arab^ (2.3%). For those with a hemoglobinopathy, 10/14 babies (71.5%) had sickle cell anemia (HbSS), 2/14(14.3%) sickle beta0 thalassemia (Sβ0 thal), 1/14 (7.1%) sickle HbC (SC) and 1/14 (7.1%) Hb DD. Thirteen out of fourteen (93%) of those with hemoglobinopathy had ethnic origin from outside Lebanon including Syria, Egypt, Turkey, Iran and Saudi Arabia.

**Table 1:** Description of the whole study population.

Parameter	% of total
**Regional Distribution**	
North	29.9%
South	25.1%
Bika'a	20%
Beirut	16.3%
Mount Lebanon	8.7%
**Total Consanguinity**	
Yes	14.8%
No	85.2%
**% Consanguinity by Region**	
North	16.6%
South	14.3%
Bika'a	19.2%
Beirut	9.6%
Mount Lebanon	12.6%
**Delivery Mode**	
Normal Vaginal	57.5%
C-section	42.5%
**Abnormal Hb**	
Yes	2.1%
No	97.9%
**Hb variant**	
HbS	84.4%
HbD	10.5%
HbC	2.8%
HbO^Arab^	2.3%

The number of neonates lost to screening was highest at the beginning of the project and in those born by normal vaginal delivery where the mother was discharged in less than 24 hours after delivery. One newborn with disease (1/14) was not screened at birth and presented to our center at 3 months of age. One hundred and forty six out of 211 (69%) of parents repeated the test within an average postnatal age of 5.2±7 months (Table 2). All repeat testing results were in agreement with the initial screening results. Thirty one percent of neonates did not undergo rescreening either because their parents refused or because they were unreachable.

**Table 2:** Description of newborns with positive screening for an abnormal hemoglobin.

Parameter	% of total
**Repeated the test**	
Yes	31%
No	69%
**Gender**	
Male	53%
Female	47%
**Consanguinity of Parents**	
Yes	28.1%
No	71.9%
**Regional Distribution**	
North	50.2%
South	25.1%
Bika'a	5.2%
Beirut	13.7%
Mount Lebanon	5.7%
**Genotype**	
Carriers	93%
Homozygous	7%

When newborns were regionally categorized according to the location of the hospital they were born at, 29.9% came from the North, 25.1% from the South, 20% from Bika'a, 16.3% from Beirut and 8.7% from Mount Lebanon (Table 1). More than 50% of newborns with a Hb variant were born in North Lebanon, while Bika'a (5.2%) had the least births with Hb variants (Table 2). Of those with a hemoglobinopathy, 13/14 (93%) had ethnic origin from outside Lebanon including Syria, Egypt, Turkey, Iran and Saudi Arabia.

Data on consanguinity, delivery mode, and birth weight were missing from 2021 (20%), 1051 (10.4%) and 1408 (13.9%) newborns, respectively (Table 1). Of those documented, 14.8% of marriages were consanguineous, 57.5% had normal vaginal deliveries and mean birth weight was 3147±473 grams. The frequency of consanguinity varied among regions. Specifically, Bika'a area had the highest rate of consanguinity (19.9%) while it was lowest in Beirut (9.6%) (Table 1). Among carriers of sickle and other Hb variants, 28.1% were born to consanguineous parents.

Newborns with normal Hb had significantly higher rate of C-section delivery compared to those with a Hb variant (χ^2^(1) = 7.066, p = 0.008). Also, newborns with sickle and other Hb variants (mean = 3254±529 grams) had significantly higher birth weight than those with normal (mean = 3145±471 grams) Hb (p<0.05) and significantly higher rates of consanguinity (28.1%) compared to newborns with normal Hb (14.6%) profile (χ^2^(1) = 21.723, p<0.005). A higher percentage of neonates with a Hb variant were born to 1st degree (58.5%) consanguineous parents compared to 3rd degree consanguinity (34.1%).

The cost of a single HPLC analysis was calculated to be $3.0. Additional costs including transport from hospitals to the screening center and printing of pamphlets and posters was estimated as $0.5/neonate making a total cost of a single screening to be $3.5.

All neonates with confirmed disease except one were enrolled in 2 free comprehensive SCD clinics in North Lebanon and central Beirut and cared for on a regular basis by a multidisciplinary team. They received penicillin, folic acid and immunizations including pneumococcal, haemophilus influenza, meningococcal and flu immunizations and were examined every 2 months and as needed. Parental education about SCD inheritance and its life threatening complications particularly acute splenic sequestration (ASS) and sepsis, as well as the need for urgent medical evaluation in cases of fever, lethargy, increasing pallor and abdominal distension was offered at diagnosis and during each clinic visit. Free genetic counseling was provided to the parents of all carriers and of those with disease.

Newborns with abnormal Hb, both heterozygote or homozygotes, had significantly higher rates of consanguinity (28.1%) compared to newborns with normal hemoglobin (14.6%) profile (χ^2^(1) = 21.723, p<0.005). As expected, homozygosity was more common in children of consanguineous compared to the rest of the population, heterozygotes and normal χ^2^(1) = 15.65, p<.001. We then explored differences among abnormal and normal Hb across degrees of consanguinity (1^st^, 2^nd^ and 3^rd^) using a Kruskal-Wallis test. The result indicated to a significant difference on across degrees of consanguinity between the two groups (χ^2^(1) = 9.91, p = .002). The resulting finding was followed up by the Mann Whitney test for tied ranks. Herein, corresponding pairs of consanguinity degrees were specified and compared across the two groups of abnormal and normal Hb. When 1^st^ and 2^nd^ degree consanguinity were compared across normal and abnormal Hb no statistically significant differences were observed (p>.05). Statistically significant differences emerged when 2^nd^ vs. 3^rd^ and 1^st^ vs. 3^rd^ degree consanguinity were compared across groups (Mann-Whitney *U* = 6973.5, p = .003 and Mann-Whitney *U* = 41625.5, p = .000, respectively). Upon exploring the distribution of degree of consanguinity across normal versus abnormal Hb, the most significant difference was observed between the 1^st^ and 3^rd^ degrees whereby a higher percentage of individuals with abnormal Hb were born to 1^st^ degree (58.5%) consanguineous parents compared to 3^rd^ degree consanguinity (34.1%) (Table 3). Moreover, a higher percentage of individuals born to 3^rd^ degree consanguinity (34.1%) had abnormal Hb as compared to those born to 2^nd^ degree consanguinity (7.3%).

**Table 3:** Abnormal hemoglobin versus Degree of Consanguinity.

			Degree			Total
			1st degree	second degree	third degree	
AbnormalHb	Positive	Count	24	3	14	41
		% within AbnormalHb	58.5%	7.3%	34.1%	100.0%
	Negative	Count	757	129	103	989
		% within AbnormalHb	76.5%	13.0%	10.4%	100.0%
Total		Count	781	132	117	1030
		% within AbnormalHb	75.8%	12.8%	11.4%	100.0%

Thirteen neonates with disease, 10 SS, 2 Sβ0, 1 SC, 7 males, 6 females, median follow up 14 months (follow up range 7 to 34 months) were closely followed for SCD related events and hospitalizations. Fourteen pain crises, 2 dactylitis episodes, 15 acute splenic sequestration events (ASS), 1 acute chest syndrome (ACS), one silent cerebral infarct (SCI), 3 acute anemic episodes not related to ASS and 41 hospitalizations were reported. The most common causes of hospitalizations were ASS (36.5%), pain crisis (34%), dactylitis (5%), acute anemic episodes (7.5%), ACS (2.5%) and viral infections (14.5%). No bacteremia or other life-threatening infections were noted. All 13 children are alive and compliant with treatment.

## Discussion

This first nationwide NBS study has shown that the incidence of sickle and other Hb variants across Lebanon was 21 per 1000 neonates (2.1%). The most common Hb variant encountered was HbS (84.4%). *HbD, HbC* and *HbO* ^Arab^ were identified for the first time in the Lebanese population. The carrier rate varied amongst regions being highest in Northern Lebanon. The incidence rate of SCD was approximately 1 in 1000 (0.1%). It is possible that there may be a risk of underestimation of this incidence due to early discharge and failure to do screening at birth. Based on our results, sickle and other Hb variants in Lebanon are as common as G6PD deficiency (2.1%) [7] and much more frequent than PKU, galactosemia and congenital adrenal hyperplasia [8]. Screening for these latter disorders has been almost a routine in Lebanon for the past 2 decades.

Compared to international data, the carrier rate of sickle and other Hb variants in Lebanon is similar to Rio de Janeiro, Brazil (approximately 2%) but almost twice that of Ferrara, Italy (1.2%) [9], [10]. Compared to Arab countries, our carrier rate is lower than Saudi Arabia (1.3–21.3%) and Bahrain (11.2–16.4%), but similar to Iraq, Jordan, Libya, Tunisia, UAE, Yemen [11]–[20].

The 0.1% incidence rate of SCD and other Hb variant disease in the Lebanese population is higher than the UAE (0.67%) and lower than the Eastern provinces of Saudi Arabia (3.8%) [11], [14], [21], [22]. Compared to other Arab countries such as Libya (0.37%), Yemen (0.9%), and Bahrain (0.6–2.1%), the incidence of the disease in Lebanon is relatively low. This may be due to the purported lesser rate of consanguineous marriages among the Lebanese population compared to other Arab countries.

Ethnic origin studies on neonates with disease suggest that the Lebanese sickle cell mutation may be the result of gene influx and migration from Africa and the surrounding Middle Eastern countries. A previous study from Lebanon determining the β-globin gene haplotypes in SCD demonstrated that 45/50 patients (90%) had haplotypes of African origin [23].

Despite the significant complications seen in the 13 followed infants, no infant has died. Early death due to infections and or ASS is not uncommon in SCD especially in SS patients. In the Jamaican newborn cohort, 13% of SS children died by 2 years of age and mostly between 6–12 months of age [24]. In the cooperative study of SCD, the mortality rate was significantly lower than in the Jamaican cohort and was highest between 6 months and 3 years of age [25]. In the UK neonatal cohort and thanks to modern treatment, no deaths were reported in the under 5-year old age group [26]. Penicillin prophylaxis, timely immunizations and education of parents about serious SCD complications and the need for prompt medical intervention coupled with availability of red blood cell transfusions and treatment in a comprehensive medical setting can explain the lack of mortality in this small Lebanese neonatal cohort.

Despite the unequivocal effectiveness of NBS in prognosticating SCD, routine screening is still unavailable in Lebanon for several reasons. The low level of genetic literacy and limited awareness about SCD and benefits of early diagnosis and treatment amongst health workers, health policy makers and parents, the social stigma of hereditary diseases, religious and cultural views, paucity of resources and presence of other competing health priorities impede promoting screening strategies in the country. The social stigmatism and religious views peculiar to Arab countries may explain why only 69% of parents of positively screened babies complied with repeating HPLC.

This first nationwide NBS program has demonstrated that sickle cell disorders represent a previously unrecognized public health burden in Lebanon and need to be handled as a major health priority. Ethnic origin studies on neonates with hemoglobinopathies suggest that the Lebanese sickle cell mutation may be the result of gene influx and migration from surrounding Middle Eastern countries. Systematic screening for SCD and other Hb variants was shown to be feasible, cost effective and of accurate predictive value. This program was also clinically effective because it led to the identification of babies with disease and to providing them with free early multidisciplinary SCD care. As the program continues to accrue neonates with disease and offer them long term comprehensive care, an improved infant mortality is expected to become similar to other modern cohorts.

The high incidence of SCD and other Hb variants in Lebanon calls for the urgent implementation of national prevention, neonatal screening and delivery of care programs through the establishment of affordable public health services. Because of the difficulty of reliably determining an infant's race by appearance, name, region or self-report, and of the expected unacceptability by the Lebanese health authorities to do targeted screening, a universal NBS screening program is recommended. Based on this study's result, amending the current Lebanese premarital health certificate to include screening for SCD is conceived by us as another urgent necessity.

Challenges of a future nationwide NBS program in Lebanon include need for more SCD awareness and acceptability of early detection programs particularly in areas of high prevalence, tracking all the new cases for immediate confirmatory testing and initiating early management. Further improvements incorporating molecular studies should obviously be achieved in order to identify neonates with beta thalassemia major, alpha thalassemia and other rare Hb variants.
